# Understanding agoraphobic avoidance: the development of the Oxford Cognitions and Defences Questionnaire (O-CDQ)

**DOI:** 10.1017/S1352465822000030

**Published:** 2022-05

**Authors:** Laina Rosebrock, Sinéad Lambe, Sophie Mulhall, Ariane Petit, Bao S Loe, Simone Saidel, Maryam Pervez, Joanna Mitchell, Nisha Chauhan, Eloise Prouten, Cindy Chan, Charlotte Aynsworth, Elizabeth Murphy, Julia Jones, Rosie Powling, Kate Chapman, Robert Dudley, Anthony Morrison, Eileen O’Regan, David M Clark, Felicity Waite, Daniel Freeman

**Affiliations:** 1 Department of Psychiatry, University of Oxford, Oxford, UK; 2 Oxford Health NHS Foundation Trust, Oxford, UK; 3 NIHR Oxford Health Biomedical Research Centre, Oxford, UK; 4 The Psychometrics Centre, University of Cambridge, Cambridge, UK; 5 Cumbria, Northumberland, Tyne and Wear NHS Foundation Trust, Newcastle upon Tyne, UK; 6 Greater Manchester Mental Health Foundation Trust, Manchester, UK; 7 Nottinghamshire Healthcare NHS Foundation Trust, Nottingham, UK; 8 Avon and Wiltshire Mental Health Partnership (AWP) NHS Trust, Bath, UK; 9 University of Newcastle, Newcastle upon Tyne, UK; 10 Division of Psychology and Mental Health, University of Manchester, Manchester, UK; 11 Department of Experimental Psychology, University of Oxford, Oxford, UK

**Keywords:** agoraphobic avoidance, cognitive model, measure development, safety-seeking behaviours, threat cognitions

## Abstract

**Background:**

Many patients with mental health disorders become increasingly isolated at home due to anxiety about going outside. A cognitive perspective on this difficulty is that threat cognitions lead to the safety-seeking behavioural response of agoraphobic avoidance.

**Aims:**

We sought to develop a brief questionnaire, suitable for research and clinical practice, to assess a wide range of cognitions likely to lead to agoraphobic avoidance. We also included two additional subscales assessing two types of safety-seeking defensive responses: anxious avoidance and within-situation safety behaviours.

**Method:**

198 patients with psychosis and agoraphobic avoidance and 1947 non-clinical individuals completed the item pool and measures of agoraphobic avoidance, generalised anxiety, social anxiety, depression and paranoia. Factor analyses were used to derive the Oxford Cognitions and Defences Questionnaire (O-CDQ).

**Results:**

The O-CDQ consists of three subscales: threat cognitions (14 items), anxious avoidance (11 items), and within-situation safety behaviours (8 items). Separate confirmatory factor analyses demonstrated a good model fit for all subscales. The cognitions subscale was significantly associated with agoraphobic avoidance (*r* = .672, *p* < .001), social anxiety (*r* = .617, *p* < .001), generalized anxiety (*r* = .746, *p* < .001), depression (*r* = .619, *p* < .001) and paranoia (*r* = .655, *p* < .001). Additionally, both the O-CDQ avoidance (*r* = .867, *p* < .001) and within-situation safety behaviours (*r* = .757, *p* < .001) subscales were highly correlated with agoraphobic avoidance. The O-CDQ demonstrated excellent internal consistency (cognitions Cronbach’s alpha = .93, avoidance Cronbach’s alpha = .94, within-situation Cronbach’s alpha = .93) and test–re-test reliability (cognitions ICC = 0.88, avoidance ICC = 0.92, within-situation ICC = 0.89).

**Conclusions:**

The O-CDQ, consisting of three separate scales, has excellent psychometric properties and may prove a helpful tool for understanding agoraphobic avoidance across mental health disorders.

## Introduction

The anxious avoidance of everyday situations such as public transport, shops or crowds – agoraphobia – occurs across many different mental health disorders. Agoraphobia symptoms are a common feature in depression (Kennedy *et al*., 2007), social anxiety (Chambless, 1985), panic disorder (Clark, 1986) and psychosis (Freeman *et al*., 2019b). A survey of 1800 patients with psychosis attending mental health services indicated that nearly two-thirds were experiencing avoidance at agoraphobia levels (Freeman *et al*., 2019b). This anxious withdrawal often persists even after the remittance of the defining symptoms of a disorder (e.g. Davidson *et al*., 2016). A cognitive perspective is that the avoidance is a safety-seeking response to threat cognitions (i.e. anticipated harm). Existing self-report measures of threat cognitions have typically been developed for an individual disorder, leading to a sole focus on content characteristic of that condition. The diagnosis of agoraphobia has been particularly linked to threat cognitions of being trapped or having a panic attack. However, anxious avoidance is likely to occur in response to a wider range of threat cognitions. In depression, avoidance of everyday situations could be due to fears of failing or being rejected (e.g. Beck, 1967); in social anxiety, due to fears of being negatively evaluated or judged (e.g. Hofmann, 2007); and in psychosis, caused by fears of being attacked (Freeman, 2016) or being unable to cope with voices (Thomas *et al*., 2015). Although certain cognitions may be characteristic of particular disorders, they are not confined to those presentations. Our aim was to develop a single brief measure capturing key cognitions that feature in many clinical disorders which may drive agoraphobic avoidance.

Avoidance can be viewed as a safety-seeking behaviour (Salkovskis, 1991; Salkovskis *et al*., 1999), an action used to prevent a feared outcome from occurring that maintains the threat cognition via the prevention of the receipt of disconfirmatory evidence. To avoid confusion in clinical work, our group calls safety-seeking behaviours ‘defences’ (Freeman, 2016), as in the treatment of paranoia we encourage the learning of safety. When the feared outcome does not occur, individuals attribute this to the success of the safety-seeking or defence action rather than that the threat cognition was incorrect. Even when situations are entered, most anxious individuals use subtle within-situation safety-seeking behaviours. For example, individuals may try to complete the task as quickly as possible (i.e. rush), increase vigilance (i.e. focus on signs of danger in the environment or identify easy exits), or limit the likelihood of a social interaction (e.g. avoid eye contact or keep distance from others). These within-situation safety-seeking behaviours are similarly used across clinical diagnoses, including depression (e.g. Newby and Moulds, 2010), anxiety disorders (Salkovskis, 1991; McManus *et al*., 2008) and psychosis (Freeman *et al*., 2001).

The aim of this study was therefore to develop a brief measure, with three separate scales, assessing (1) key cognitions that may drive agoraphobic avoidance, (2) anxious avoidance of common everyday situations, and (3) within-situation safety-seeking behaviours. Importantly, the measure was designed to assess a broad range of threat cognitions so that it can be used across clinical disorders.

## Method

### Participants

We recruited three participant groups: non-clinical individuals (*n* = 612) scoring above the Mobility Inventory (MI) threshold for the presence of agoraphobia (≥2.3); non-clinical individuals (*n* = 1335) scoring below the MI threshold for the presence of agoraphobia; and patients with psychosis (*n* = 198) being treated for agoraphobic avoidance as part of a clinical trial. See Table 1 for a summary of demographic characteristics.

**Table 1.** Participant demographic and clinical characteristics

	Patients with psychosis (*n* = 198)	Low agoraphobia (*n* = 1335)	High agoraphobia (*n* = 612)
Age (mean, *SD*)	36.51 (12.77)	48.54 (15.79)	44.13 (15.09)
Age range	16–71	18–84	18–80
Gender	*n* (%)	*n* (%)	*n* (%)
Male	136 (68.7%)	341 (25.5%)	136 (22.2%)
Female	61 (30.8%)	976 (73.1%)	458 (74.8%)
Non-binary/other	0	16 (1.2%)	16 (2.6%)
Prefer not to say	0	2 (0.1%)	2 (0.3%)
Ethnicity			
White	171 (86.4%)	1269 (95.1%)	580 (94.8%)
Asian (any)	4 (2%)	17 (1.3%)	8 (1.3%)
Black (any)	4 (2%)	8 (0.6%)	1 (0.2%)
Mixed/multiple/other	18 (9.1%)	28 (2.1%)	17 (2.6%)
Prefer not to say	0	12 (0.9%)	6 (1.0%)
Diagnosis			
Schizophrenia	80 (41.0%)	—	—
Psychosis not otherwise specified	79 (40.0%)	—	—
Schizoaffective disorder	11 (5.6%)	—	—
Bipolar affective disorder	6 (3.1%)	—	—
Depression with psychotic features	18 (9.2%)	—	—
Delusional disorder	2 (1.0%)	—	—
Age in first contact with services (mean, *SD*)	25.62 (10.67)	—	—

The patients with psychosis were recruited as part of gameChange, a multi-site randomised controlled trial of an automated virtual reality intervention to reduce agoraphobic avoidance (Freeman *et al*., 2019c). The main inclusion criteria for the gameChange trial are: (1) age 16 years+, (2) a diagnosis of non-affective or affective psychosis, and (3) self-reported difficulties going into everyday situations due to anxiety.

Non-clinical participants were recruited using advertisements on social media. The inclusion criteria were: 18 years or older and residing in the United Kingdom.

### Measures

#### Oxford Cognitions and Defences Questionnaire (O-CDQ; Supplementary Appendix 1)

An item pool of 46 items was developed (see Table 2), corresponding to three distinct subscales (threat cognitions, anxious avoidance, and within-situation safety behaviours). The item pool consisted of 14 threat cognition items, 22 anxious avoidance items, and 10 within-situation safety behaviours (i.e. defences). The threat cognition items were chosen to correspond to seven different symptoms (depression, social anxiety, panic disorder, persecutory ideation, ideas of reference, aggression, and verbal hallucinations), with two items per presentation. The anxious avoidance and within-situation safety behaviour items included common places that people with agoraphobia typically avoid (e.g. cafés, shops) and strategies used to reduce anxiety in those situations (e.g. avoiding eye contact), respectively. The items were generated by four cognitive clinical psychologists using these guiding principles, a review of existing measures for several individual mental health disorders, and an analysis of responses in previous studies from patients with psychosis, Participants were asked to rate for the past fortnight how often they had a specific cognition, how frequently they had avoided situations, and how often they had used within-situation safety behaviours. Each item was rated on a 4-point scale from 0 (never) to 3 (always), with higher ratings indicating greater severity.

**Table 2.** Oxford Cognitions and Defences Questionnaire (O-CDQ) original item pool

**Threat cognitions**
1. I will embarrass myself
2. I will fail
3. People will judge me negatively
4. I will be rejected
5. I will panic
6. I will lose control
7. Everyone will watch me
8. People will laugh at me
9. I will become verbally aggressive
10. People will try to upset me
11. I will physically harm someone else
12. People will harm me physically
13. I won’t be able to cope with voices
14. Voices will harm me in some way
**Anxious avoidance**
1. My local shop
2. Shopping centres
3. Supermarkets
4. Using public transport (e.g. bus, train)
5. Pubs
6. Restaurants
7. My neighbours
8. Strangers
9. GP surgery or health centre
10. Cafés
11. Gyms
12. Walking on the street
13. Meeting people or social gatherings
14. People in authority (e.g. the police)
15. My workplace or place of education
16. Open spaces
17. Enclosed spaces
18. Staying at home alone
19. Staying at home with others
20. Being far from home
21. Unfamiliar places
22. Busy places
**Within-situation safety behaviours**
1. I avoided making eye contact
2. I only went out if someone I know was with me
3. I watched out for signs that something bad might happen
4. I left as soon as I started to feel anxious
5. I scanned faces for signs of judgement or criticism
6. When out, I kept my distance from other people
7. I formed an escape plan
8. I spent excessive time thinking about what bad things might happen
9. When out, I did everything as quickly as possible
10. I listened out for trouble

#### Mobility Inventory for Agoraphobia (MI; Chambless *et al.,* 1985)

The MI is a self-report measure assessing current avoidance of situations due to anxiety (i.e. agoraphobia) when alone. Items ask about avoidance of places (e.g. restaurants, car parks), transportation (e.g. buses, trains), riding/driving in a car, and specific situations (e.g. walking on the street, standing in queues). Each item is rated on a 5-point scale, from 1 (never avoid) to 5 (always avoid) and there is an option to select ‘not applicable’ (n/a). The average score is derived from all items that were not answered ‘n/a’. Higher average scores indicate higher levels of agoraphobia. An average score of 2.3 and above is considered the clinical cut-off (National Collaborating Centre for Mental Health, 2020).

#### Oxford Agoraphobic Avoidance Scale (O-AS; Lambe *et al.*, 2021)

The O-AS is a new 8-item scale assessing agoraphobic avoidance and anxiety in everyday situations. Items concern a range of everyday activities (e.g. travelling on a bus for several stops, sitting in a café for 10 minutes). Participants are asked to provide two ratings: whether they think they could do the task now (avoidance rating) and how anxious they would feel completing the task (distress rating). Higher scores on both the avoidance and distress scale indicate greater difficulties.

#### Generalised Anxiety Disorder Scale-7 (GAD-7; Spitzer *et al.*, 2006)

The GAD-7 is a 7-item scale assessing symptoms of generalised anxiety in the past two weeks. Example items include ‘feeling nervous, anxious or on edge’ and ‘feeling afraid as if something awful might happen’. Each item is rated on a 4-point scale, from 0 (not at all) to 3 (nearly every day). Higher scores indicate higher levels of generalised anxiety.

#### Brief Fear of Negative Evaluation Scale (BFNE; Leary, 1983)

The BFNE is a 12-item scale assessing fear of being negatively evaluated. Example items include ‘I am usually worried about what kind of impression I make’ and ‘I am afraid that people will find fault with me’. Items are rated on a 5-point scale, from 1 (not at all characteristic of me) to 5 (extremely characteristic of me). Four items are reverse-coded. Higher scores indicate higher levels of concerns with being negatively evaluated by others.

#### Patient Health Questionnaire-9 (PHQ-9; Kroenke *et al.*, 2001)

The PHQ-9 is a 9-item self-report questionnaire measuring symptoms of depression over the past two weeks (e.g. feeling down; difficulty concentrating; psychomotor retardation/agitation). Each of the items corresponds to a diagnostic criteria item for major depression. Items are rated on a 4-point scale, from 0 (not at all) to 3 (nearly every day). Higher scores indicate higher levels of depression.

#### Revised Green et al. Paranoid Thoughts Scale (R-GPTS; Freeman *et al.*, 2019a)

The R-GPTS is an 18-item self-report questionnaire assessing paranoid thinking over the past two weeks. It contains two subscales: ideas of reference (10 items) and persecution (8 items). Example items for each subscale include ‘I spent time thinking about friends gossiping about me’ and ‘I was sure someone wanted to hurt me’. Each item is rated on a 5-point scale, from 0 (not at all) to 4 (totally). Higher scores indicate higher levels of paranoid thinking.

### Procedure

All participants completed the O-CDQ item pool, MI, R-GPTS and PHQ-9. The non-clinical participants completed the GAD-7 and the BFNE. Patients with psychosis also completed the O-AS.

Non-clinical participants completed all measures online. Patients with psychosis completed the measures either in person with the support of a research assistant, online, via post, or over the telephone (remote completion was implemented for participants after September 2020 due to the COVID-19 pandemic).

To evaluate test–re-test reliability, 372 non-clinical participants repeated the O-CDQ after two weeks.

### Statistical analysis

Analyses were conducted in R, version 4.0.3 (R Core Team, 2020) and SPSS, version 26.0 (IBM, 2020). Exploratory and confirmatory factor analyses (EFA and CFA) were used to derive the final set of items for the O-CDQ. CFA was conducted using the ‘lavaan’ package (Rosseel, 2012). We divided the participants into two groups: a derivation sample and a validation sample. The derivation sample included 75% of the combined group of non-clinical individuals scoring above the MI threshold for presence of agoraphobia (score ≥2.3) and patients with psychosis. This consisted of 464 non-clinical participants with high levels of agoraphobia and 143 patients with psychosis (combined *n* = 607). EFA was conducted with the derivation sample. The validation sample included the remaining 25% of the combined group of non-clinical individuals scoring above the MI threshold for presence of agoraphobia and patients with psychosis, and all non-clinical individuals scoring below the MI threshold for presence of agoraphobia. This consisted of 148 non-clinical individuals with high levels of agoraphobia, 55 patients with psychosis, and 1335 non-clinical individuals with low levels of agoraphobia (combined *n* = 1538). CFA was conducted with the validation sample.

Given the small number of items for the threat cognitions subscale and the pre-specified theoretically driven factor structure, for this subscale only a CFA in the validation sample was run initially (using the 7-factor model with two items per factor) with the variance of all factors fixed to 1 for identification reasons. Although preferable to have more than two items per factor, it is possible to retain factors with two items if the factor loadings are high (above 0.70) and the between-factor correlations are moderate (e.g. Worthington and Whittaker, 2006; Yong and Pearce, 2013). Additionally, we examined other test theory methods (e.g. internal consistency and test–re-test reliability) to further support the use of the model.

Separate EFAs were conducted prior to CFA for the other two subscales (anxious avoidance and within-situation safety behaviours). Items that were either highly correlated (*r*≥0.8) or had a low correlation with other items (did not have correlations above 0.3 with any other item) were deleted prior to EFA. EFA was estimated using principal axis factoring and oblique rotation, as we expected the factors to correlate. Parallel analysis and inspection of scree plots were used to determine the number of factors to extract, following the criteria of retaining only factors with eigenvalues above 1. Items were considered for deletion based on factor loadings (did not load onto any factor above 0.30, or had cross-loadings above 0.30 on multiple factors), communalities ( < 0.30) and content (e.g. theoretically inconsistent or redundant). Once a final set of items was derived via EFA, CFA was conducted for the avoidance and within-situation behaviours subscales separately to assess the model fit in the validation sample. The MLR robust maximum likelihood estimator was used to account for skewness of the data. Good model fit was determined using Hu and Bentler’s (1999) recommended thresholds of above 0.95 on the comparative fit index (CFI) and Tucker–Lewis index (TLI), below 0.10 on the root mean square of approximation (RMSEA), and below 0.08 on the standardized root mean square residual (SRMR).

Pearson correlations were used to examine convergent validity. One-way ANOVAs were used to examine differences in cognitions, avoidance, and within-situation behaviours between the psychosis group, non-clinical individuals with agoraphobic avoidance, and non-clinical individuals without agoraphobic avoidance. Test–re-test reliability was run using the ‘irr’ package in R.

## Results

### Threat cognitions subscale

The initial CFA of the 14-item, 7-factor model (with two items per factor) indicated an excellent model fit (χ^2^ = 295.93, d.f. = 56, *p* < .001, CFI = .970, TLI = .951, RMSEA = .053, SRMR = .031) with almost all factor loadings above 0.70 (except for items 11 and 12, which were 0.69 and 0.65, respectively). See Table 3 for factor loadings. Factor correlations are also provided in Supplementary Table 1. Furthermore, we conducted CFA examining a higher-order model to identify the existence of a global latent factor (i.e. threat cognitions). This indicated an acceptable model fit (χ^2^ = 618.68, d.f. = 70, *p* < .001, CFI = .931, TLI = .910, RMSEA = .071, SRMR = .064). A total threat cognitions score, calculated by summing all the items, could therefore be used for subsequent validity analyses. Factor loadings for the higher-order model are provided in Supplementary Table 2.

**Table 3.** Final items and factor loadings from exploratory factor analysis (EFA) and confirmatory factor analysis (CFA)

Threat cognitions	CFA loadings
**Depression**	
1. I will embarrass myself	0.812
2. I will fail	0.842
**Social anxiety**	
3. People will judge me negatively	0.913
4. I will be rejected	0.842
**Panic**	
5. I will panic	0.857
6. I will lose control	0.846
**Social reference**	
7. Everyone will watch me	0.881
8. People will laugh at me	0.886
**Harming others**	
9. I will become verbally aggressive	0.774
11. I will physically harm someone else	0.693
**Persecution**	
10. People will try to upset me	0.759
12. People will harm me physically	0.651
**Voices**	
13. I won’t be able to cope with voices	0.896
14. Voices will harm me in some way	0.826

Anxious avoidance	EFA loadings	CFA loadings
**Shopping/being around others**		
1. My local shop	0.573	0.768
2. Shopping centres	0.851	0.866
3. Supermarkets	0.877	0.871
4. Using public transport (e.g. bus, train)	0.541	0.739
12. Walking on the street	0.510	0.718
**Social places/meeting others**		
7. My neighbours	0.655	0.688
9. GP surgery or health centre	0.705	0.648
10. Cafés	0.480	0.794
13. Meeting people or social gatherings	0.549	0.757
14. People in authority (e.g. the police)	0.696	0.675
15. My workplace or place of education	0.487	0.649

Within-situation safety behaviours	EFA loadings	CFA loadings
**Avoiding others**		
1. I avoided making eye contact	0.539	0.680
4. I left as soon as I started to feel anxious	0.571	0.761
6. When out, I kept my distance from other people	0.796	0.849
9. When out, I did everything as quickly as possible	0.687	0.783
**Hypervigilance**		
3. I watched out for signs that something bad might happen	0.891	0.854
5. I scanned faces for signs of judgement or criticism	0.413	0.726
7. I formed an escape plan	0.577	0.735
10. I listened out for trouble	0.777	0.862

Cronbach’s alphas indicated high levels of internal consistency for the total scale (.93) and all subscales: depression (.82), social anxiety (.88), panic (.86), social reference (.89), harming others (.67), persecution (.70) and voices (.87).

### Anxious avoidance subscale

Factor analysis for the anxious avoidance subscale was appropriate as Bartlett’s test of sphericity was significant (χ^2^ = 5912.44, d.f. = 231, *p* < .001) and the Kaiser–Myer–Olkin (KMO) test of sampling adequacy was high (KMO = .92). No items were deleted prior to EFA. During EFA, 11 items were deleted: four items had communalities below 0.30 (‘Enclosed spaces’, ‘Staying at home alone’, ‘Staying at home with others’, ‘Gyms’), five items were vaguely worded and/or did not fit with other items in their factor (‘Busy places’, ‘Strangers’, ‘Being far from home’, ‘Unfamiliar places’, ‘Open spaces’), and two items were deleted due to very high factor loadings suggesting that they were driving the existence of that factor (‘Restaurants’, ‘Pubs’). The scree plot and parallel analysis test of the remaining 11 items indicated that a two-factor model was the most appropriate fit for the data, with a factor correlation of 0.64. Factors were identified as ‘Shopping/Being around others’ and ‘Social places/Meeting others’. This model explained 43.4% of the variance.

A CFA with the validation sample was conducted using the two-factor 11-item model from EFA. This indicated an excellent model fit (χ^2^ = 218.15, d.f. = 43, *p* < .001, CFI = .961, TLI = .951, RMSEA = .051, SRMR = .030). Factor loadings of the final items are presented in Table 3. The between-factor correlation was 0.886. Additionally, a total anxious avoidance score, calculated by summing all the items, was used for subsequent validity analyses as a bi-factor model similarly indicated an excellent model fit (χ^2^ = 112.74, d.f. = 33, *p* < .001, CFI = .982, TLI = .971, RMSEA = .040, SRMR = .019). Factor loadings for the bi-factor model are provided in Supplementary Table 3.

Cronbach’s alpha indicated high internal consistency for the total scale (.94) and both subscales: shopping (.92) and social places (.88).

### Within-situation safety behaviours subscale

Factor analysis for the within-situation safety behaviours subscale was appropriate as Bartlett’s test of sphericity was significant (χ^2^ = 2393.95, d.f. = 34, *p* < .001) and the KMO test of sampling adequacy was high (KMO = .90). No items were deleted prior to EFA. During EFA, two items were deleted: one had communalities below 0.30 (‘I only went out if someone I knew was with me’), and one was judged to be worded too vaguely (‘I spent excessive time thinking about what bad things might happen’). The scree plot and parallel analysis test of the remaining eight items indicated that a two-factor model was the most appropriate fit for the data, with a factor correlation of 0.79. Factors were identified as ‘Self-protection’ and ‘Hypervigilance’. This model explained 46.5% of the variance.

A CFA with the validation sample was conducted using the two-factor, 8-item model from EFA. This indicated a good model fit (χ^2^ = 193.69, d.f. = 19, *p* < .001, CFI = .959, TLI = .940, RMSEA = .077, SRMR = .036). Factor loadings of the final items are presented in Table 3. The between-factor correlation was 0.832. As with the threat cognitions and anxious avoidance subscales, a total within-situation score, calculated by summing all the items, was used for subsequent validity analyses as a bi-factor model similarly indicated an excellent model fit (χ^2^ = 92.24, d.f. = 12, *p* < .001, CFI = .981, TLI = .956, RMSEA = .066, SRMR = .019). Factor loadings for the bi-factor model are provided in Supplementary Table 4.

Cronbach’s alpha indicated high internal consistency for the total scale (.93) and both subscales: avoiding others (.88) and hypervigilance (.88).

### Validity

The O-CDQ threat cognition subscale was significantly correlated with both the O-CDQ anxious avoidance (*r* = .716, *p* < .001) and within-situation safety behaviours (*r* = .799, *p* < .001) subscales.

#### Correlations with agoraphobic avoidance

The threat cognition subscale was significantly positively correlated with the MI (*r* = .673, *p* < .001), the O-AS avoidance subscale (*r* = .518, *p* < .001) and the O-AS distress subscale (*r* = .676, *p* < .001). The O-CDQ anxious avoidance subscale was significantly positively correlated with agoraphobic avoidance as measured by both the MI (*r* = .867, *p* < .001) and the O-AS avoidance (*r* = .709, *p* < .001) and distress (*r* = .822, *p* < .001) subscales. Lastly, the OCDQ within-situation safety behaviours subscale was positive correlated with agoraphobic avoidance measured by the MI (*r* = .757, *p* < .001) and the O-AS avoidance (*r* = .574, *p* < .001) and distress (*r* = .752, *p* < .001) subscales.

#### Correlations with other mental health outcomes

Additionally, the O-CDQ threat cognition subscale was significantly positively correlated with the GAD-7 (*r* = .746, *p* < .001), the BFNE (*r* = .617, *p* < .001), the PHQ-9 (*r* = .619, *p* < .001), the R-GPTS ideas of reference subscale (*r* = .754, *p* < .001) and the R-GPTS persecutory ideation subscale (*r* = .655, *p* < .001).

#### Group differences for each subscale

Mean scores for each of the three subscales for each group (non-clinical individuals without agoraphobic avoidance, non-clinical individuals with agoraphobic avoidance, and patients with psychosis) are given in Table 4. One-way ANOVAs indicated that there were significant differences between the non-clinical individuals and the patients with psychosis in levels of threat cognitions (*F*_2,2142_ = 520.301, *p* < .001), anxious avoidance (*F*_2,2142_ = 1356.02, *p* < .001) and within-situation safety behaviours (*F*_2,2142_ = 839.634, *p* < .001). Bonferroni-corrected multiple comparisons indicated that individuals without agoraphobic avoidance had the lowest scores on all subscales and non-clinical individuals with agoraphobic avoidance had lower scores than patients with psychosis for both the anxious avoidance and within-situation safety behaviours subscales but not the threat cognitions subscale.

**Table 4.** Mean scores and standard deviations for each O-CDQ subscale by participant group

O-CDQ subscale	Mean (*SD*)
**Threat cognitions total score**	
Patients with psychosis (*n* = 198)	19.19 (8.76)^a^
High agoraphobia (*n* = 612)	18.84 (8.38)^a^
Low agoraphobia (*n* = 1335)	8.10 (6.91)^b^
**Anxious avoidance total score**	
Patients with psychosis (*n* = 198)	18.07 (8.56)^a^
High agoraphobia (*n* = 612)	15.28 (7.18)^b^
Low agoraphobia (*n* = 1335)	3.16 (3.89)^c^
**Within-situation safety behaviours total score**	
Patients with psychosis (*n* = 198)	14.59 (5.42)^a^
High agoraphobia (*n* = 612)	13.24 (5.94)^b^
Low agoraphobia (*n* = 1335)	4.46 (4.45)^c^

a>b, b>c at *p*<.01.

### Reliability

Test–re-test reliability was excellent for all scales: threat cognitions (*n* = 372, ICC = 0.88, 95% CI: 0.85–0.90), anxious avoidance (*n* = 372, ICC = 0.92, 95% CI: 0.90–0.93) and within-situation safety behaviours (*n* = 372, ICC = 0.89, 95% CI: 0.87–0.91).

## Discussion

A core tenet of cognitive theory is that cognitions drive unhelpful behavioural responses. This provides a powerful approach in understanding and treating many mental health disorders. One unhelpful response, agoraphobic avoidance, driven by fear cognitions, is a common feature across many mental health problems. This paper reports the development of the Oxford Cognitions and Defences Questionnaire (O-CDQ, Supplementary Appendix 1), designed for researchers and clinicians to quickly assess and understand the key cognitions and behaviours common to agoraphobic difficulties across clinical disorders. Factor analyses demonstrated an excellent model fit for the subscales assessing threat cognitions, anxious avoidance, and within-situation safety-seeking behaviours (i.e. defences). All subscales had high reliability. Each subscale consists of several factors but also comprises a general latent factor. Therefore, total scores for each subscale are used, making the scale easy to understand and administer. A total score from the three subscales is not meaningful and is not used. Assessed in a large participant group, the psychometric properties of the three scales are strong.

The three subscales of the O-CDQ – threat cognitions, anxious avoidance, and within-situation safety-seeking behaviours – were all highly correlated. This suggests that the measure is capturing the link between fearful cognitions (e.g. ‘I will fail’, ‘I will embarrass myself’, ‘Everyone will watch me’) and use of behaviours aimed at reducing the feared outcome from occurring. Importantly, the cognitions subscale was highly associated with two validated measures of agoraphobic avoidance. This subscale was also highly associated with measures of depression, social anxiety, generalized anxiety and paranoia. This suggests that this subscale may be useful in understanding key cognitions across these clinical presentations to inform formulation and link to interventions that directly target these cognitions. The avoidance and within-situation subscales were also highly correlated with existing measures of agoraphobic avoidance, supporting their validity. Furthermore, there were significant differences in scale scores between participants with and without agoraphobia symptoms.

There are several key limitations. The non-clinical participants were recruited online using social media and therefore will not be representative of the general population. The non-clinical participant group with high levels of agoraphobic avoidance was determined using a self-report scale rather than clinical interview. The patient group was limited to individuals with psychosis. Although patients with psychosis typically have high levels of anxiety and depression, future studies could test the scale in other diagnostic groups. Test–re-test data were only collected for the non-clinical group. Lastly, the cognitions subscale consisted of factors comprising two items. Although two-item factors are inferior to standard multi-item factors, the items were theoretically driven and the subscale demonstrated high internal consistency and test–re-test reliability, as well as excellent convergent validity. The O-CDQ is being used in the multi-centre gameChange clinical trial, and its true value will become apparent.

## Data Availability

All requests for access to the de-identified data will be considered by the Chief Investigator of the study and team. The intent is to share data for reasonable requests. Data will be made available to external researchers subject to the constraints of the consent under which data were collected, with an appropriate data sharing agreement.

## Supplementary material

For supplementary material accompanying this paper visit https://doi.org/10.1017/S1352465822000030. click here to view supplementary material
