# Supplementary material for: Understanding agoraphobic avoidance: the development of the Oxford Cognitions and Defences Questionnaire (O-CDQ)
Source: Behav Cogn Psychother. 2022 Feb 15;50(3):257–68. doi: 10.1017/S1352465822000030 (PMC9378026; doi:10.1017/S1352465822000030)
Supplement: Supplementary file 1 [file S1352465822000030sup.zip › S1352465822000030sup001.docx]

Appendix 1: The Oxford Cognitions and Defences Questionnaire (O-CDQ)

## **Part 1: Fearful thoughts about being outside (worries):**

People sometimes find it difficult to go into everyday situations because they worry that something bad will happen. This questionnaire asks about the worries you may have before going out, or when you are outside your home.

Please read each statement below and select the number that best describes how much you’ve had this fear over the **past two weeks.**

| In the past two weeks I feared that: | | Never | Occasionally | Often | Always |
| --- | --- | --- | --- | --- | --- |
| 1. | I will embarrass myself. | 0 | 1 | 2 | 3 |
| 2. | I will fail. | 0 | 1 | 2 | 3 |
| 3. | People will judge me negatively. | 0 | 1 | 2 | 3 |
| 4. | I will be rejected. | 0 | 1 | 2 | 3 |
| 5. | I will panic. | 0 | 1 | 2 | 3 |
| 6. | I will lose control. | 0 | 1 | 2 | 3 |
| 7. | Everyone will watch me. | 0 | 1 | 2 | 3 |
| 8. | People will laugh at me | 0 | 1 | 2 | 3 |
| 9. | I will become verbally aggressive. | 0 | 1 | 2 | 3 |
| 10. | I will physically harm someone else. | 0 | 1 | 2 | 3 |
| 11. | People will try to upset me. | 0 | 1 | 2 | 3 |
| 12. | People will harm me physically. | 0 | 1 | 2 | 3 |
| 13. | I won’t be able to cope with voices. | 0 | 1 | 2 | 3 |
| 14. | Voices will harm me in some way. | 0 | 1 | 2 | 3 |

## **Part 2: Keeping away from outside situations (avoidance):**

Fears and worries often lead to people not going into situations. They avoid situations because of the fears of what may happen. Please rate how often you have avoided the situations below in the **past two weeks** as a result of worries.

| In the past two weeks I have avoided: | | Never | Occasionally | Often | Always |
| --- | --- | --- | --- | --- | --- |
| 1. | My local shop | 0 | 1 | 2 | 3 |
| 2. | Shopping Centres | 0 | 1 | 2 | 3 |
| 3. | Supermarkets | 0 | 1 | 2 | 3 |
| 4. | Using public transport (e.g. bus, train) | 0 | 1 | 2 | 3 |
| 5. | Walking on the street | 0 | 1 | 2 | 3 |
| 6. | My neighbours | 0 | 1 | 2 | 3 |
| 7. | GP surgery or health centre | 0 | 1 | 2 | 3 |
| 8. | Cafes | 0 | 1 | 2 | 3 |
| 9. | Meeting people or social gatherings | 0 | 1 | 2 | 3 |
| 10. | People in authority (e.g. the police) | 0 | 1 | 2 | 3 |
| 11. | My workplace or place of education | 0 | 1 | 2 | 3 |

**Part 3: Dealing with risks when outside (defences):**

There may be times when a person can't avoid being in situations that worry them. At these times they may try to reduce the risk. They may put up defences to keep themselves safe.

Please circle the number that best describes how often you may have used each strategy to try and keep yourself safe in the **past two weeks.**

| In the past two weeks: | | Never | Occasionally | Often | Always |
| --- | --- | --- | --- | --- | --- |
| 1. | I avoided making eye contact. | 0 | 1 | 2 | 3 |
| 2. | I left as soon as I started to feel anxious. | 0 | 1 | 2 | 3 |
| 3. | When out, I kept my distance from other people. | 0 | 1 | 2 | 3 |
| 4. | When out, I did everything as quickly as possible. | 0 | 1 | 2 | 3 |
| 5. | I watched out for signs that something bad might happen. | 0 | 1 | 2 | 3 |
| 6. | I scanned faces for signs of judgement or criticism. | 0 | 1 | 2 | 3 |
| 7. | I formed an escape plan. | 0 | 1 | 2 | 3 |
| 8. | I listened out for trouble. | 0 | 1 | 2 | 3 |
