# Supplementary material for: Understanding agoraphobic avoidance: the development of the Oxford Cognitions and Defences Questionnaire (O-CDQ)
Source: Behav Cogn Psychother. 2022 Feb 15;50(3):257–68. doi: 10.1017/S1352465822000030 (PMC9378026; doi:10.1017/S1352465822000030)
Supplement: Supplementary file 1 [file S1352465822000030sup.zip › S1352465822000030sup002.docx]

**Supplementary Table 1**

*Factor Correlations for the Threat Cognitions Subscale of the O-CDQ from Confirmatory Factor Analysis (CFA)*

|  | Depression | Social Anxiety | Panic | Social Reference | Harming Others | Persecution |
| --- | --- | --- | --- | --- | --- | --- |
| Depression | - |  |  |  |  |  |
| Social Anxiety | 0.937*** | - |  |  |  |  |
| Panic | 0.784*** | 0.753*** | - |  |  |  |
| Social Reference | 0.823*** | 0.856*** | 0.754*** | - |  |  |
| Harming Others | 0.420*** | 0.411*** | 0.533*** | 0.398*** | - |  |
| Persecution | 0.644*** | 0.733*** | 0.751*** | 0.740*** | 0.749*** | - |
| Voices | 0.394*** | 0.428*** | 0.509*** | 0.489*** | 0.473*** | 0.622*** |

****p*<.001

**Supplementary Table 2**

*Factor Loadings from Higher-Order Confirmatory Factor Analysis (CFA) Model – Threat Cognitions Subscale*

| **Threat Cognitions** | Factor Loadings – Items | Higher Order Factors |
| --- | --- | --- |
| Depression | - | 0.933*** |
| 1. I will embarrass myself. | 0.817*** | - |
| 1. I will fail. | 0.837*** | - |
| Social Anxiety | - | 0.948*** |
| 1. People will judge me negatively. | 0.906*** | - |
| 1. I will be rejected. | 0.849*** | - |
| Panic | - | 0.840*** |
| 1. I will panic. | 0.869*** | - |
| 1. I will lose control. | 0.834*** | - |
| Social Reference | - | 0.896*** |
| 1. Everyone will watch me. | 0.881*** | - |
| 1. People will laugh at me. | 0.886*** | - |
| Harming Others | - | 0.501*** |
| 1. I will become verbally aggressive. | 0.845*** | - |
| 1. I will physically harm someone else. | 0.635*** | - |
| Persecution | - | 0.790*** |
| 1. People will try to upset me. | 0.820*** | - |
| 1. People will harm me physically. | 0.603*** | - |
| Voices | - | 0.513*** |
| 1. I won’t be able to cope with voices. | 0.909*** | - |
| 1. Voices will harm me in some way. | 0.814*** | - |

****p*<.001

**Supplementary Table 3**

*Factor Loadings from Bi-factor Confirmatory Factor Analysis (CFA) Model – Anxious Avoidance Subscale*

| **Anxious Avoidance** | Specific Factors | General Factor |
| --- | --- | --- |
| Shopping/Being around others |  |  |
| 1. My local shop | 0.025 | 0.775*** |
| 1. Shopping centres | 0.374* | 0.820*** |
| 1. Supermarkets | 0.344 | 0.824*** |
| 1. Using public transport (e.g. bus, train) | 0.030 | 0.748*** |
| 1. Walking on the street | -0.098 | 0.758*** |
| Social places/Meeting others |  |  |
| 1. My neighbours | 0.411*** | 0.598*** |
| 1. GP surgery or health centre | 0.293*** | 0.581*** |
| 1. Cafes | 0.161* | 0.768*** |
| 1. Meeting people or social gatherings | 0.350*** | 0.677*** |
| 1. People in authority (e.g. the police) | 0.348*** | 0.597*** |
| 1. My workplace or place of education | 0.303*** | 0.582*** |

Note: **p*<.05, ****p*<.001. When running bi-factor models, it is not unusual to observe low, negative, and non-significant factor loadings for specific factors (e.g. Gomez, Stavropoulous, & Griffiths, 2020). This suggests that the general factor accounts for a larger part of the variance than both specific factors, indicating the scale can be used to report a total sum score. Nonetheless, there is still clinical relevance in having two separate factors, which the CFA also supports. Therefore, either a total sum score or individual factor scores (e.g. for shopping and social places) can be interpreted.

**Supplementary Table 4**

*Factor Loadings from Bi-factor Confirmatory Factor Analysis (CFA) Model – Within-Situation Safety Behaviours Subscale*

| **Within-Situation Safety Behaviours** | Specific Factors | General Factor |
| --- | --- | --- |
| Avoiding Others |  |  |
| 1. I avoided making eye contact. | 0.196* | 0.656*** |
| 1. I left as soon as I started to feel anxious. | -0.147 | 0.795*** |
| 1. When out, I kept my distance from other people. | 0.396 | 0.853*** |
| 1. When out, I did everything as quickly as possible. | -0.027 | 0.782*** |
| Hypervigilance |  |  |
| 1. I watched out for signs that something bad might happen. | 0.520*** | 0.680*** |
| 1. I scanned faces for signs of judgement or criticism. | 0.218*** | 0.697*** |
| 1. I formed an escape plan. | 0.369*** | 0.629*** |
| 1. I listened out for trouble. | 0.581*** | 0.680*** |

Note: **p*<.05, ****p*<.001. When running bi-factor models, it is not unusual to observe low, negative, and non-significant factor loadings for specific factors (e.g. Gomez, Stavropoulous, & Griffiths, 2020). This suggests that the general factor accounts for a larger part of the variance than both specific factors, indicating the scale can be used to report a total sum score. Nonetheless, there is still clinical relevance in having two separate factors, which the CFA also supports. Therefore, either a total sum score or individual factor scores (e.g. avoiding others and hypervigilance) can be interpreted.

REFERENCES

**Gomez, R., Stavropoulos, V. & Griffiths, M. D.** (2020). Confirmatory factor analysis and exploratory structural equation modelling of the factor structure of the Depression Anxiety and Stress Scales – 21. *PLoS One, 15*(6), e0233998. doi: 10.1371/journal.pone.0233998.
